# Fear of COVID-19 when experiencing pregnancy or childbirth in the pandemic: what are the associated factors?

**DOI:** 10.1590/0034-7167-2022-0755

**Published:** 2023-12-08

**Authors:** Ana Júlia de Paula, Paulo César Condeles, Jéssica Aparecida da Silva, Luciano Marques dos Santos, Luciana Mara Monti Fonseca, Mariana Torreglosa Ruiz, Monika Wernet

**Affiliations:** I Universidade Federal do Triângulo Mineiro. Uberaba, Minas Gerais, Brazil; II Universidade Estadual de Feira de Santana. Feira de Santana, Bahia, Brazil; III Universidade de São Paulo. Ribeirão Preto, São Paulo, Brazil; IV Universidade Federal de São Carlos. São Carlos, São Paulo, Brazil

**Keywords:** COVID-19, Pregnancy, Postpartum Period, Fear, Pandemics, COVID-19, Embarazo, Periodo Posparto, Miedo, Pandemias, COVID-19, Gravidez, Período Pós-Parto, Medo, Pandemias

## Abstract

**Objective:**

to identify factors associated with fear of COVID-19 among women who experienced pregnancy or childbirth during the pandemic.

**Methods:**

a cross-sectional study, nested within a prospective cohort, using an online survey, from August 2021 to February 2022, based on descriptive data analysis.

**Results:**

of the 431 participants, 52.8% were postpartum women and 20.1% were pregnant women. With regard to fear of COVID-19, a mean score of 20.46 was obtained (moderate fear). The highest fear scores were present in women whose newborns were admitted to hospital in neonatal critical units (p=0.032), and the lowest among those covered by supplementary health (insurance) (p=0.016).

**Conclusion:**

among pregnant and postpartum women, high fear of COVID-19 translated into the possibility of having newborns admitted to hospital in a critical unit. The importance of supporting actions to support pregnant/postpartum women’s mental health in relation to COVID-19 or other threats that may influence the neonatal outcome stands out.

## RESUMO

**Objetivo:**

identificar fatores associados ao medo da COVID-19 entre mulheres que vivenciaram a gestação ou parto durante a pandemia.

**Métodos:**

estudo transversal aninhado à coorte prospectiva, por meio de inquérito online, no período de agosto de 2021 a fevereiro de 2022, a partir de análise descritiva dos dados.

**Resultados:**

dos 431 participantes, 52,8% eram puérperas e 20,1% gestantes. Com relação ao medo da COVID-19, obteve-se pontuação média de 20,46, (medo moderado). Os maiores escores de medo estiveram presentes em mulheres cujos recém-nascidos estavam internados em unidades críticas neonatais (p = 0,032) e os menores entre cobertos pela saúde suplementar (convênios) (p = 0,016).

**Conclusão:**

Entre gestantes e puérperas o alto medo da COVID-19 traduziu-se na possibilidade de ter o neonato internado em unidade crítica. Destaca-se a importância de subsidiar ações de suporte à saúde mental de gestantes/puérperas, com relação à COVID-19 ou outras ameaças, que possam influenciar o desfecho neonatal.

## RESUMEN

**Objetivo:**

identificar factores asociados al miedo al COVID-19 entre mujeres que vivieron el embarazo o el parto durante la pandemia.

**Métodos:**

estudio transversal, anidado dentro de una cohorte prospectiva, mediante encuesta en línea, de agosto de 2021 a febrero de 2022, basado en análisis de datos descriptivos.

**Resultados:**

de los 431 participantes, el 52,8% eran puérperas y el 20,1% eran gestantes. En cuanto al miedo al COVID-19 se obtuvo una puntuación media de 20,46 (miedo moderado). Las puntuaciones de miedo más altas estuvieron presentes en las mujeres cuyos recién nacidos fueron hospitalizados en unidades críticas neonatales (p=0,032), y las más bajas entre las cubiertas por el seguro complementario de salud (p=0,016).

**Conclusión:**

entre las mujeres embarazadas y puérperas, el alto temor a la COVID-19 se tradujo en la posibilidad de internar al recién nacido en una unidad de críticos. Se destaca la importancia de apoyar acciones para apoyar la salud mental de las mujeres embarazadas/en posparto en relación con el COVID-19 u otras amenazas que puedan influir en el resultado neonatal.

## INTRODUCTION

Worldwide, more than 750 million cases of infection with SARS-CoV-2 (Severe Acute Respiratory Syndrome Coronavirus 2), the causative agent of COVID-19 (Coronavirus Disease 2019), have been recorded. More than 6 million deaths occurred from the disease^(1)^, which was declared a pandemic by the World Health Organization (WHO) in 2020. The high lethality of COVID-19 demanded quick responses and important adaptations for its containment, especially with regard to the fields of research, development of immunobiological agents and health care.

In Brazil, pregnant and postpartum women accounted for approximately 1% of cases of infection, with 24,280 reported cases, of which 2,057 (8.5%) died^(2)^, i.e., an index considered very high when compared to international data (1.8%)^(3)^. In this regard, studies by the Obstetric Observatory pointed out that maternal deaths in Brazil were 35% higher than the rates reported by the WHO^(2)^. The data, extracted from an interactive public data analysis monitoring platform (health, socioeconomic and environmental), were scientifically grounded with regard to the dissemination of relevant information in the area of maternal and child health, with state and municipal cuts^(2)^.

Pregnant women and postpartum women, due to the physiological changes of pregnancy, are predisposed to infection by the coronavirus, with an increased risk of developing severe forms of COVID-19^(4, 5, 6)^, aspect that led to considering them among the priority risk groups for assistance and testing for COVID-19 in Brazil and worldwide^(4, 5)^.

Literature on COVID-19 and pregnancy indicated an increase in the percentage of cesarean sections due to maternal respiratory decompensation and/or fetal hypoxemia^(3, 7, 8, 9, 10, 11, 12, 13)^; increase in premature births^(3, 7, 9, 10, 13, 14, 15, 6)^; occurrence of more severe cases with rapid progression to pneumonia^(9, 13, 14, 15)^; increase in abortions and stillbirths^(16)^ and maternal deaths^(16)^, emphasizing that, specifically in Brazil, the number of maternal deaths secondary to COVID-19 was notorious^(17)^. It is noteworthy that a meta-analysis pointed out that the worst maternal and neonatal outcomes during the pandemic occurred in low- and middle-income countries^(16)^. Moreover, it is noteworthy that women with previous comorbidities or pathologies acquired during pregnancy had a higher risk of unfavorable outcomes^(18)^.

During the physiological pregnancy-puerperal cycle, women undergo important physical, hormonal and psychological changes, which can enhance feelings of anxiety, insecurity and fear^(19)^. More specifically, fear of infection by the coronavirus was among the main causes of hesitation for assistance in the general population^(20)^ as well as among pregnant women^(17)^. A study pointed out that perceiving COVID-19 as a serious threat and with higher fear scores resulted in an increase in the adoption of protective measures^(21)^. Hesitation for assistance, especially during pregnancy, can compromise the outcome of COVID-19 as well as pregnancy prognosis.

Thus, it is necessary to investigate fear of the disease, the behaviors and associated factors. Among the revisited bibliographies, a study that investigated the applicability and validity of a scale that measures fear of COVID-19^(20)^, in its Brazilian Portuguese version stands out, including a random sample of 1,734 Brazilians, which showed higher anxiety and fear scores among women^(22)^. The same behavior was pointed out in a meta-analysis that observed higher fear scores among women^(23)^; however, both studies did not measure this variable in the obstetric population. Furthermore, another study with 209 pregnant women pointed out that anxiety, as a frequent sensation during pregnancy, especially in the third trimester of pregnancy, it is also related to the presence and/or fear of gestational complications and their outcomes, including childbirth^(24)^.

Added to these characteristic aspects of the pregnancy-puerperal period, the COVID-19 pandemic may have further heightened fear in this specific population, not only with regard to the possibility of infection, but the need for assistance and, in cases that were associated with the infection, the possible complications that may result in the outcome of the pregnancy itself. Thus, this study is justified by elucidating the factors associated with high fear of COVID-19 in women who experienced pregnancy or childbirth during the pandemic period.

Given the above, this study proposed a look at fear of COVID-19 in the Brazilian obstetric population.

## OBJECTIVE

To identify factors associated with fear of COVID-19 among women who experienced pregnancy or childbirth during the pandemic.

## METHODS

### Ethical aspects

The study was conducted in accordance with national and international ethics guidelines, approved by the Research Ethics Committee, whose opinion is attached to this submission, and its entire development was guided and guided by the Guidelines and Regulatory Norms for Research involving human beings, contained in Resolution 466/12/CNS/MoH.

### Study design, place and period

This is a cross-sectional study nested within a prospective cohort using an online survey, with data collected between August 2021 and February 2022.

### Population and sample

Social media followers who interacted with content related to the research theme were invited to the study, mostly women with experience of being pregnant during the pandemic and who followed the profiles created on social media, or even those invited by followers (snowball technique). Thus, the study included people who had access to the internet, aged over 18 years and who, consenting to participate in the research, filled out the online form. The sample is non-probabilistic for convenience.

### Study protocol

The data collection form was prepared by the researchers themselves, built in the Hyper Text Markup Language (HTML) standard on Google Forms® and sent for validity by three experts in the area, respecting all ethical criteria. Literature recommendations were followed for validity, whose prerogatives indicate from six to twenty validators, with a minimum of three individuals, when this represents a professional group^(25)^, as in the case of this sample. The three experts were specialists in obstetric and neonatal nursing, one working in a tertiary care hospital, one in a childbirth home, and one in teaching; of these, two were masters and one was a doctor. The form was validated with 100% agreement, in its second version, after adjustments in wording, preserving content.

Consent terms were prepared both for validators and study participants, adapting objectives and language, according to each category, and, in both cases, submission was carried out online. At the end of the home page, after reading the term, participants could tick the options: 1 – I have read and consent to participate; 2 – I have read and do not consent to participate (in this case, the survey was closed). If they consented, participants were directed to the data collection form.

The virtual environment of social networks of an extension project constituted the scenario of this study. In mid-July 2020, a group of partner researchers from the *Universidade Federal do Triângulo Mineiro* (UFTM) and *Universidade Federal de São Carlos* (UFSCar) carried out studies in the thematic area of COVID-19 in pregnancy, childbirth and the puerperium, translating and disseminating knowledge in social networks created for this purpose. Named “@nascer.e.covid”/”*Nascer e Covid*”, on Instagram® and Facebook®, the extension project made more than 200 posts so that its page on Instagram® counts, in December 2022, with more than 2,800 followers and, on Facebook®, with 225 followers.

The invitation to the study was posted on these networks, with the provision of a link to the Informed Consent Form. Upon consenting to participate in the study, respondents were directed to a questionnaire with sociodemographic data. Later, when selecting pregnant women’s or postpartum women’s opinion, they were directed to a specific questionnaire, which they filled in with information on clinical and obstetric data as well as on occurrence of COVID-19. If they responded affirmatively to the disease, they were also directed to a form about it. In the case of postpartum women, information about the birth and the child was also asked.

To measure fear of COVID-19 infection, the translated and validated version of the Fear of COVID-19 Scale was used, which was kindly provided by Faro *et al*.^(26)^. The Fear of COVID-19 Scale must be self-completed and has seven statements related to the infection. Five alternative Likert-type responses are presented, ranging from 1 to 5, with 1 being the parameter marked when strongly disagreeing with the proposition and 5 when strongly agreeing. At the end, all marked items are added together with their respective weight (1 to 5). Scores between 7 and 19 points classify as little fear; from 20 to 26 points indicate moderate fear; and participants who total scores above 27 have a high fear score of COVID-19^(26, 27)^.

### Analysis of results, and statistics

Sampling was done for convenience during the data collection period. The dependent variable of the study was the high fear of COVID-19 score (score above 27 points). Sociodemographic, clinical, obstetric and COVID-19 data were investigated.

Data collected through Google Forms® were imported into a Microsoft Excel® spreadsheet and then into the Statistical Package for the Social Sciences version 23.0. Initially, descriptive analyzes (frequency, mean, standard deviation, minimum and maximum) of variables were performed, and the results were presented in tables. Chi-square and Fisher’s exact tests were applied, considering a significance level of 5%. Prevalence ratios and respective 95% Confidence Intervals were estimated.

Poisson regression with robust variance was applied in the multiple analysis, being indicated for analysis of count data and to minimize the effects of overestimation of prevalence ratio that occur when the outcome is common or very frequent in the sample^(28)^. The model was used in which independent variables were inserted in chunks in the following order: sociodemographic data; clinical data; and obstetric data. Variables with a value of < 0.20 in univariate analyzes were included in the model. Variable selection in the model was performed using the backward stepwise method. By this method, all variables with a value equal to 0.20 in the univariate analysis are considered for the Wald statistic in maintaining the variables during the level-by-level adjusted analysis, in order to control potential confounding factors^(28, 29)^ and identify real association factors.

## RESULTS

Thus, 431 participants responded to the survey, 96.7% of whom were female. Among respondents, 52.8% were postpartum women; 20;1% were pregnant; 12.7% were undergraduate students in the health area; 10.8% were health professionals; and 3.6% reported being family members of pregnant women or postpartum women.

The mean age was 30.96 (6.54 years), ranging from 18 to 61 years. The majority declared themselves to be white (65.1%), married (54.3%), that they had a high level of education with at least higher education (63.3%), that they perform paid activities (76.9%), that they reside in the Southeast region (68.7%), with a predominance of residents in the state of Minas Gerais (63.4%). Most reported having health insurance (74.2%), but they used the Brazilian Health System (*Sistema Único de Saúde*) (56.6%). In the obstetric population, it is noteworthy that the average number of pregnancies was 1.69 (0.93 pregnancies), ranging from zero to five.

Among the participants, 45.1% (188) referred to fear of COVID-19; about 35.3% (147) indicated moderate fear; and 19.7% (82) indicated high fear. The average score was 20.46 ± 6.30, indicating moderate fear. It is noteworthy that, of the 431 participants, 417 (96.8%) responded to the fear of infection form. The total score ranged from seven to 35 points (maximum score).

In the bivariate analysis, the association of sociodemographic variables and clinical conditions with high fear of COVID-19 (scores above 27 points) in the general population is presented in Table 1. A statistically significant association was observed between the variable “having health insurance” and high fear of COVID-19 (p=0.016), in which participants covered by supplementary health had lower fear scores. Other variables in the general population were not statistically significant in relation to high fear of COVID-19, as shown in Table 1.

**Table 1:** Association between sociodemographic variables and clinical conditions with high fear of COVID-19 in the general population, Brazil, 2022

Variable among general population	High fear of COVID-19		Did not have a high fear of COVID-19		PR	CI	p value*
	n	%	n	%			
Female	78	19.4	324	80.6	1.128	(0.807-1.577)	0.490
Male	4	28.6	10	71.4			
White	47	17.2	227	82.8	0.701	(0.475-1.033)	0.091
Non-white	35	24.5	108	75.5			
Has paid occupation	55	18.1	249	81.9	0.810	(0.518-1.266)	0.370
Does not have paid occupation	21	22.3	73	77.7			
Has health insurance	51	16.6	256	83.4	0.593	(0.400 – 0.878)	0.016
Does not have health insurance	30	28.0	77	72.0			
SUS user	47	20.0	188	80.0	1.040	(0.702-1.540)	0.901
Non-SUS user	35	19.2	147	80.8			
Postpartum women	47	21.9	168	78.1	1.212	(0.819-1.794)	0.387
Non-postpartum women	35	18.1	159	81.9			
Pregnant	13	16.0	68	84.0	0.763	(0.444-1.310)	0.356
Non-pregnant	69	21.0	259	79			
Obstetric population	60	20.3	236	79.7	1.041	(0.672- 1.613)	0.891
General population	22	19.5	91	80.5			
Undergraduate student	12	22.6	41	77.4	1.151	(0.671-1.976)	0.585
Non-undergraduate student	70	19.7	286	80.3			
Health professional	5	11.1	40	88.9	0.525	(0.225-1.229)	0.165
Non-health professional	77	21.2	287	78.8			
Family member of obstetric population	5	33.3	10	66.7	1.706	(0.811-3.586)	0.194
Non-family member obstetric population	77	19.5	317	80.5			

Note: PR - Prevalence Ratio; CI - 95% Confidence Interval; SUS - Brazilian Health System; *Chi-square and Fisher’s exact tests.

The bivariate analysis between high fear of COVID-19 and variables of the obstetric population showed a statistically significant relationship between the variable newborn (NB) forwarded to the Intensive Care Unit (ICU) or Intermediate Care Unit (InCU) with high fear of COVID-19, indicating that women/mothers who had their children admitted to hospital in these units had a high fear of COVID-19 (p= 0.032). The other variables associated with the obstetric population and neonatal outcomes are shown in Table 2, but no statistical significance was found.

**Table 2:** Association between obstetric variables and clinical conditions of newborns with high fear of COVID-19 in the obstetric population, Brazil, 2022

Variable among obstetric population	High fear of COVID-19		Did not have a high fear of COVID-19		PR	CI	p value*
	n	%	n	%			
Primigravida	26	18.6	114	81.4	0.795	(0.488-1.295	0.428
Multigravida	25	23.4	82	76.6			
Infected by COVID-19	14	18.4	62	81.6	0.884	(0.511-1.529)	0.737
Non-infected by COVID-19	40	20.8	152	79.2			
Needed hospital admission due to COVID-19	1	25.0	3	75.0	1.365	(0.233-7.992)	0.571
Did not need hospital admission due to COVID-19	13	18.3	58	81.7			
Chronic disease carrier	13	28.9	32	71.1	1.603	(0.937-2.744)	0.104
Non-chronic disease carrier	40	18.0	182	82.0			
Obese	9	28.1	23	71.9	1.502	(0.812-2.777)	0.237
Non-obese	44	18.8	191	81.2			
Chronic hypertense	0	0.0	8	100.0		(1.182-1.337)	0.363
Non-chronic hypertense	53	20.5	206	79.5	1.257		
Asthmatic	5	33.3	10	66.7	1.750	(0.819-3.740)	0.187
Non-asthmatic	48	19.0	204	81.0			
Developed disease during pregnancy	11	22.4	38	77.6	1.138	(0.634-2.043)	0.695
Did not develop disease during pregnancy	43	19.7	175	80.3			
Developed gestational diabetes	7	21.8	25	78.2	1.094	(0.542-2.209)	0.816
Did not develop gestational diabetes	47	20.0	188	80.0			
Developed gestational hypertension	4	23.5	13	76.5	1.176	(0.482-2.871)	0.756
Did not develop gestational hypertension	50	20.0	200	80.0			
Developed hypothyroidism	2	50.0	2	50.0	2.529	(0.921-6.942)	0.183
Did not develop hypothyroidism	52	19.8	211	80.2			
Vaccinated against COVID-19	39	21.4	143	78.6	1.607	(0.429-6.015)	0.741
Non-vaccinated against COVID-19	2	13.3	13	86.7			
Normal childbirth	17	22.7	58	77.3		(0.854-1.170)	0.564
Cesarean section	26	22.6	89	77.4	0.999		
Premature NB	4	28.6	10	71.4	0.776	(0.324-1.857)	0.524
Non-premature NB	39	22.1	137	77.9			
Low weight NB	5	26.3	14	73.7	0.860	(0.385-1.918)	0.774
Non-low weight NB	38	22.6	130	77.4			
NB forwarded to ICU/InCU	6	50.0	6	50.0	2.365	(1.254-4.458)	0.032
NB not forwarded to ICU/ InCU	37	21.1	138	78.9			

Note: PR - Prevalence Ratio; CI - 95% Confidence Interval; NB – newborn; ICU - Intensive Care Unit; InCU - Intermediate Care Unit; *Chi-square and Fisher’s exact tests.

All variables with p-values <0.20 in the bivariate analysis were placed in the Poisson robust regression model (having health insurance – p= 0.016; white skin color – p=0.091; pregnant woman with a chronic disease – p= 0.104; asthmatic pregnant women p=0.187; gestational hypothyroidism – p=0.183). The variable having been infected by COVID-19 was inserted in the model, as it is the variable of interest in the study. Table 3 presents standard error, (95%) Confidence Intervals and p-values. It is noted that participants who declared themselves white showed almost significance; however, the fact of having their NB forwarded to a critical unit justified the high fear of COVID-19 in the obstetric population.

**Table 3:** Poisson regression model with robust variance between high fear of COVID-19 and sociodemographic and health variables. Brazil, 2022

Variable	PR	95%CI	p value*
With health insurance	1.062	−0.027-0.148	0.174
NB forwarded to ICU/InCU	0.837	−0.358-0.003	0.054
White	1.083	−0.002-0.162	0.055
Chronic disease carrier	0.974	−0.133-0.080	0.622
Asthmatic	0.997	−0.176-0.171	0.977
Developed hypothyroidism	0.813	−0.530-0.115	0.207
Infected by COVID-19	0.982	−0.098-0.063	0.666

*Note: PR - Prevalence Ratio; 95%CI - 95% Confidence Interval; NB – newborn; ICU - Intensive Care Unit; InCU - Intermediate Care Unit.*

## DISCUSSION

Pregnant women and postpartum women had scores that indicated moderate fear of COVID-19 (20.46). Compared to a study that used the same scale to assess fear of COVID-19 among 215 Iranian pregnant women, similar values were identified in the total score (20.85)^(27)^. However, a cross-sectional study carried out in Peru, with 449 participants, showed higher average scores for the general population (24.04), with a significant difference among women (25.90)^(30)^. This same study showed a high fear of COVID-19 in most participants (59.24%)^(30)^, unlike the results presented. But it should be noted that this study was carried out with a population that lived in conditions of social disadvantage, in addition to the fact that the collection was carried out between August and September 2020, a period of great pandemic peak.

Another study conducted in Poland with 262 pregnant women also revealed higher scores for fear of COVID-19, regardless of parity (22.33 to 23.66). The authors identified a significant association between stress, fear of COVID-19 and fear of the outcome (childbirth). Fear of infection mediated stress and increased fear of childbirth. According to them, the experience of pregnancy in the pandemic was accompanied by a negative emotional impact, as it increased not only fear of infection, but the stress and fear of childbirth, with childbirth being seen as a threat to the mother’s and her child’s health^(31)^. It should be noted that these increased scores can also be attributed to the data collection period, which took place at the beginning of the pandemic, between March and April 2020.

Still on fear of COVID-19, among pregnant and postpartum women, a study with 184 Italian participants showed low fear (15.0) in the obstetric population, contrasting the results presented. However, although the scores are lower, the study observed that 27% had a high fear of infection, a higher rate than that found in this and other studies. Thus, the authors pointed out that, during the pandemic, women in the perinatal period, regardless of prior mental health history, experienced increased levels of anxiety, fear and psychological distress, which were attributed to isolation, quarantine, lockdown and deprivation of their support network and social contacts, not just the infection itself^(32)^.

The Chinese population study should also be highlighted, which showed high rates of depression indicators among participants and the association of symptoms with moderate fear related to COVID-19. It was also observed that women who gave birth during the pandemic were more likely to have depression and that, during the pandemic, there was an increase in post-childbirth depression rates. The authors warn that women with high fear of infection were the most vulnerable to presenting depressive symptoms in the puerperium^(33)^.

Moreover, a Canadian study with more than 9,500 pregnant women showed moderate to high scores of fear of infection. Fear of COVID-19 was associated with sociodemographic factors, such as being food insecure, belonging to certain ethnic groups and depending on the geographic location in which they resided. It was also associated with clinical factors, such as having already presented some form of anxiety before gestational diagnosis, having a chronic health condition and pre-gestational obesity, in addition to factors related to pregnancy, such as parity and, also, the stage of pregnancy at the time of the interview (third trimester). Higher fear scores were associated with anxiety and depression^(34)^. However, the authors pointed out that, similarly to the study carried out, the women had a high level of education, which limits the generalization of results. It is worth noting that, similarly, lower fear scores were found in participants who claimed to be users of the supplementary service, showing the relevance of social aspects in the experience of fear.

High fear associated with COVID-19 resulted in increased compliance with protective measures against infection^(21)^. However, the response to fear and, consequently, to the stress generated, is an individual and multifactorial characteristic. The experience can trigger coping strategies, but there can also be denial, naturalization or poor adaptation, which can result in harmful health behaviors (hyperactivity, consumption of licit and illicit drugs, changes in mental health)^(34)^.

Participants who declared themselves white showed almost significance, however the fact of having their NB forwarded to a critical unit justified fear of COVID-19 in the obstetric population. Contrasting the results, a study carried out in Turkey with 906 postpartum women showed that fear of COVID-19 was associated with socioeconomic variables such as high level of education, high income and paid activity^(35)^. However, in this same study, it was found that women undergoing cesarean section reported high fear of COVID-19, indicating that hospital admission and interventions can influence and increase the feeling of fear^(36)^, similarly to the result that pointed to the association between high fear and NB admission.

Thus, fear associated with having their NB admitted to hospital in a critical unit can be justified by the fact that NBs are in a hospital unit, but not only for this reason. Among the various reasons, fear of contaminating children who have immunological fragility, fear of becoming infected during visits as well as fear of being caused by the experience itself and the emotional critical moment experienced can be suggested. In this regard, a qualitative study with mothers of babies forwarded to the ICU pointed out that, at the time of the news, most postpartum women experience negative feelings, such as worry and fear, aggravated when this hospital admission was unexpected and unforeseen. Positive feelings arise with living together over the days and favorable evolution of infants after the state of compliance^(37)^.

With regard to nursing care for the obstetric population during the pandemic, a study pointed out that midwife-nurses led the movement to meet women’s specificities and, at the same time, reorient practices to ensure safety during the pandemic, focusing on humanized care, but based on scientific evidence^(38)^.

Thus, midwife-nurses must participate in various stages of the process, such as reorienting flows, redesigning protocols, organizing spaces for isolation, attending meetings with the unit’s management and train workers, among others, in order to guarantee the protection of the health of women, NBs, family members and the team itself^(38)^.

However, permeating the issues related to infection containment, nurses had to experience additional concerns in order to offer safe care, but at the same time, a positive birth experience for women and their families, striving for quality, humanization and scientific basis. With rapid and constant changes in care to meet the demands imposed by the disease, the process was crossed by adaptations, fears and anxieties that also compromised these professionals’ mental health^(38)^. Thus, it is observed that fear of COVID-19 permeated not only pregnant women, but also postpartum women, their families as well as health professionals and, more specifically, the nursing team, whose role was essential in providing care during the pandemic.

Finally, special attention should be paid to postpartum women, in order to guarantee the necessary resources to face the situation, which is a real threat to their mental health. Considering the above, opening space to thematize fears and concerns in perinatal practices is urgent, with emphasis on pandemic scenarios or similar situations. Dialoguing is a care strategy with therapeutic and educational development, with special attention to offering sensitive listening and welcoming manifestations of fear. This implies recognizing and valuing the woman and the interactional spaces in prenatal, childbirth and post-childbirth care.

### Study limitations

As limitations of this study, we can mention its study design, whose current moment of health crisis may not match the feeling experienced throughout pregnancy, in addition to the fact that pregnant/postpartum women who died from the disease (greater severity cases) did not make up the case series. The possibility of recall bias is also highlighted, since the data were based on participants’ responses.

Another limitation is the study collection period, which coincided with the introduction of immunization against COVID-19 for pregnant women, which may also have influenced the results and, consequently, reduced fear scores in this population. Another important limitation is the profile of participants, who were women with access to the internet, mostly with high education, with paid formal employment, white, residing in the Southeast region and users of supplementary health (health insurance). Therefore, this profile may not reflect other Brazilian women’s reality. The fact can be explained by the fact that it is a study with online collection, which limits universal participation. Furthermore, the fact that it was an online survey did not allow the severity of the cases to be classified. Another point for discussion is that the study took place in a population with high purchasing power, with access to adequate food, medication, differentiated health services, information, which may have contributed to better results regarding the infection and the pregnancy itself.

Despite these limits, the online collection, due to sanitary conditions, made it possible to present the profile of the disease in a national sample and with a cross-sectional design, which consisted of an innovation for the moment experienced. Thus, this study emerges as potential for further research, with other designs, which seek to elucidate factors that may compromise the outcome of pregnancies, such as new infections and changes in mental health due to fear of potentially threatening situations, such as the experience of having a child admitted to a critical unit.

### Contributions to nursing and health

This research can contribute to other studies, enriching new discussions on the subject. Moreover, it points to the need for actions to support pregnant and/or postpartum women’s mental health, whether in the face of COVID-19 or in the face of any threat to the pregnancy-puerperal cycle. The importance of support/support for women who have the possibility or who actually have their children admitted to critical care units and the primary role of nurses in this support is highlighted. These needs must be addressed through resources and investments in public policies as well as care team commitment to provide humanized and quality care.

## CONCLUSIONS

Our study pointed out that having supplementary health coverage, through insurance or private care, reduced fear of COVID-19. Among pregnant and postpartum women, high fear was translated into the possibility or experience of being separated from their child due to their hospital admission in a critical unit (ICU/InCU). No other factors associated with fear of infection were identified, either among pregnant and postpartum women or in the general population; however, the sample may have been insufficient to find statistical differences among the compared groups. With this, our study points to the need to carry out further investigations on the subject as well as in the face of new infections or possible threatening situations that cause fear, such as the experience of having a child admitted to hospital in a critical unit. It also reinforces the importance of support for women who experience situations of fear or anguish in the puerperal-pregnancy cycle and investments in public policies for women’s mental health.
